# Saddle Pulmonary Embolism Secondary to Nitrous Oxide Use: A Case Report and Literature Review

**DOI:** 10.7759/cureus.95353

**Published:** 2025-10-24

**Authors:** Carlos Valladares, Carlos Nuñez, Erick Villacis, Danny Allauca, Sofia Simaluisa, Maria Humbria

**Affiliations:** 1 Pulmonary and Critical Care, University of Miami Miller School of Medicine, Jackson Memorial Hospital, Miami, USA; 2 Faculty of Medical Sciences, National Autonomous University of Honduras, Tegucigalpa, HND; 3 Faculty of Medical Sciences, Catholic University of Santiago de Guayaquil, Guayaquil, ECU; 4 Faculty of Health Sciences "Eugenio Espejo", Universidad Tecnológica Equinoccial (UTE), Quito, ECU; 5 Faculty of Health Sciences, Francisco de Miranda National Experimental University, Falcon, VEN

**Keywords:** homocysteine levels, oral anticoagulation, pulmonary embolism (pe), recreational nitrous oxide use, vitamin b12

## Abstract

Saddle pulmonary embolism (SPE) is a rare but life-threatening presentation of venous thromboembolism (VTE). Recreational nitrous oxide (N₂O) use has become increasingly common and can cause a functional vitamin B12 deficiency that leads to elevated homocysteine levels and a prothrombotic state. We describe a 31-year-old man with ongoing N₂O abuse who presented with dyspnea and calf pain and was found to have a saddle pulmonary embolus and right leg deep vein thrombosis. Laboratory studies showed low vitamin B12 and high homocysteine levels. He was treated with anticoagulation and discharged in stable condition. This case highlights the need to consider N₂O exposure in patients with unexplained thrombosis, particularly in younger adults.

## Introduction

Saddle pulmonary embolism (SPE) is an uncommon anatomical subtype of acute pulmonary embolism (PE), characterized by a large visible thrombus lodged at the bifurcation of the main pulmonary artery, often extending into both pulmonary arteries, and accounts for approximately 2-5% of all PE cases [1]. It can lead to acute hemodynamic collapse, such as cardiac arrest, cardiogenic shock, and respiratory failure, representing a life-threatening medical emergency [2]. 

Nitrous oxide (N₂O) is an inhaled anesthetic gas used in dental procedures and recreationally for its euphoric and hallucinogenic effects [3]. It is most frequently used among young adults aged 18-25 years [4-5]. The recreational use of N₂O has increased in recent years: in 2021, the Global Drug Survey ranked it as the 14th most commonly used recreational drug worldwide [6], and in 2022, the European Monitoring Centre for Drugs and Drug Addiction (renamed the European Union Drugs Agency) recognized it as one of the most prevalent psychoactive substances in Europe [7].

Recreational N₂O abuse has been linked to adverse effects due to the disruption of vitamin B12 activity, leading to inactivation of its cofactors for methionine synthase and methylmalonyl-CoA mutase. Moreover, the accumulation of homocysteine secondary to methionine synthase deficiency has been proposed as a mechanism that predisposes to thromboembolic complications and increased risk of cardiovascular disease and cerebrovascular events. Nevertheless, the exact mechanisms by which N₂O exposure precipitates these events remain unclear. Available evidence is largely limited to isolated case reports and small case series, with no clinical trials to establish a robust causal relationship [8].

This case is noteworthy because it describes a rare presentation of SPE in a young adult without traditional risk factors following recreational N₂O use. Given the growing prevalence of N₂O consumption worldwide and the potential for severe thromboembolic complications in otherwise low-risk individuals, reporting this case contributes to raising clinical awareness and expanding the limited body of literature available on this topic. We accompany this report with a literature review to contextualize our findings and summarize the pathophysiological mechanisms proposed to date. The patient’s history of a prior PE and ongoing N₂O use raised concern for an underlying hyperhomocysteinemic state; however, outpatient testing after his previous hospitalization excluded genetic causes, supporting N₂O-induced functional vitamin B12 deficiency as the likely mechanism.

## Case presentation

A 31-year-old male with a past medical history of PE one year prior and N_2_O substance use disorder presented to the emergency department with complaints of shortness of breath and right calf pain for the past four days. He had been treated with apixaban for three months following his previous pulmonary embolism and was not taking any other medications at the time of presentation. He denied any fever, cough, chest pain, recent travel, or hospitalization. He endorsed ongoing recreational N_2_O use. On examination, vital signs showed a blood pressure of 124/95 mmHg, heart rate of 115 beats per minute, respiratory rate of 22, and oxygen saturation of 91% on room air. His physical examination was remarkable only for right calf tenderness without erythema, swelling, or edema, with a Well's score of 7.5 and 1, respectively. The rest of the systemic examination was unremarkable.

Initial laboratory work-up was obtained and is summarized in Table 1. Key results included elevated D-dimer, markedly increased homocysteine, and low B12 levels. Electrocardiography demonstrated sinus tachycardia.

**Table 1:** Initial laboratory results on presentation AST: aspartate aminotransferase; ALT: alanine aminotransferase; INR: international normalized ratio; aPTT: activated partial thromboplastin time

Test	Result	Reference range
On admission		
Hemoglobin	13.1 g/dL	13.5–17.5 g/dL
Hematocrit	39.8%	41–53%
White blood cell count	9.4 ×10⁹/L	4.5–11 ×10⁹/L
Platelet count	210 ×10⁹/L	150–450 ×10⁹/L
Creatinine	0.9 mg/dL	0.6–1.2 mg/dL
AST	24 U/L	10–40 U/L
ALT	29 U/L	7–56 U/L
INR	1.0	0.8–1.2
aPTT	30 sec	25–35 sec
D-dimer	2,480 ng/mL	<500 ng/mL
Arterial pH	7.38	7.35–7.45
pCO₂	39 mmHg	35–45 mmHg
pO₂	68 mmHg	80–100 mmHg
Additional testing following admission		
Homocysteine	134 µmol/L	5–15 µmol/L
Vitamin B12	142 pg/mL	200–900 pg/mL
Folate	5.8 ng/mL	2–20 ng/mL

**Figure 1:**
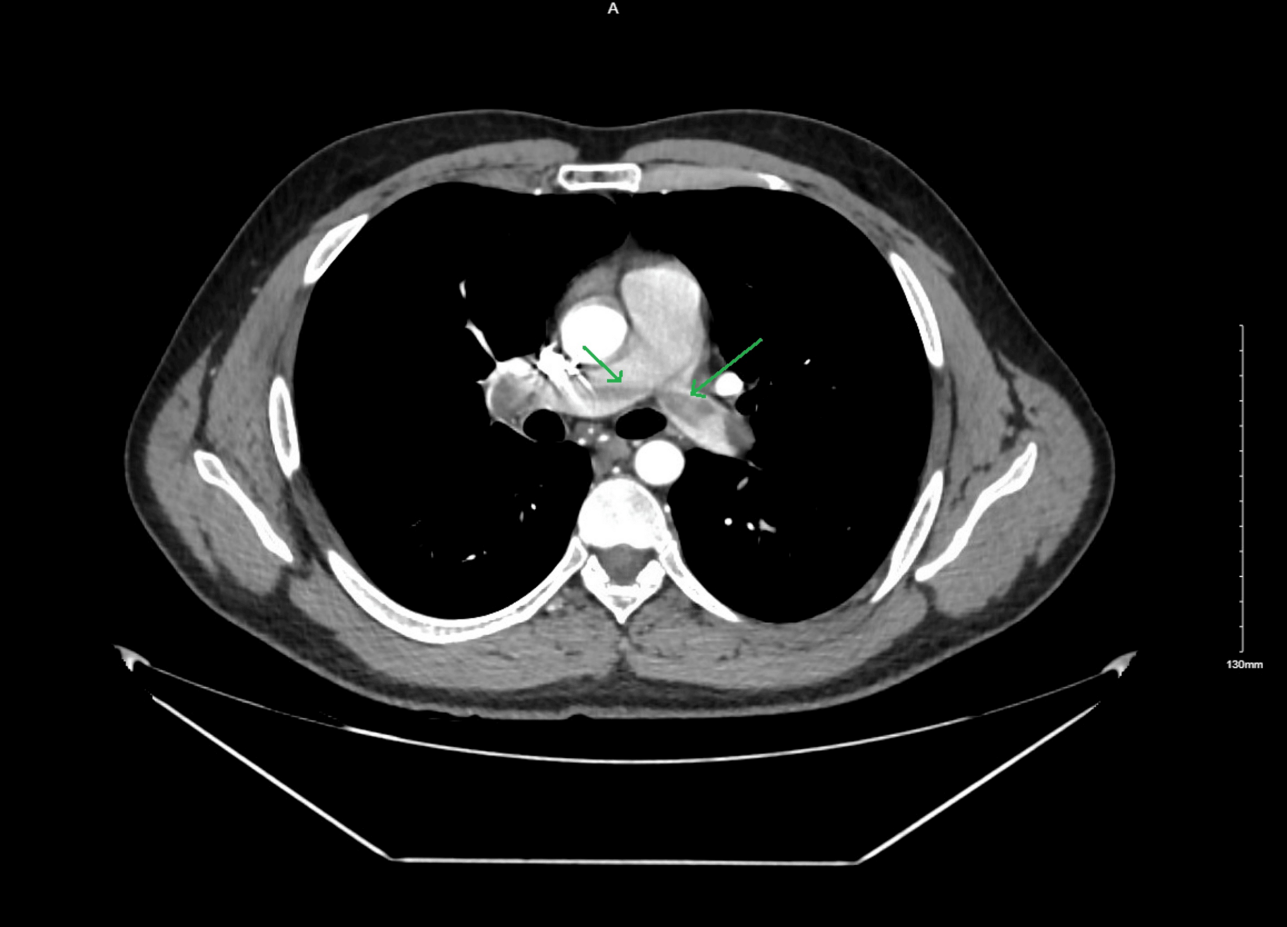
Computed tomography angiography (CTA) of the chest showing a saddle pulmonary embolism. A large thrombus is visualized at the bifurcation of the main pulmonary artery with extension into both the right and left pulmonary arteries (green arrows).

**Figure 2:**
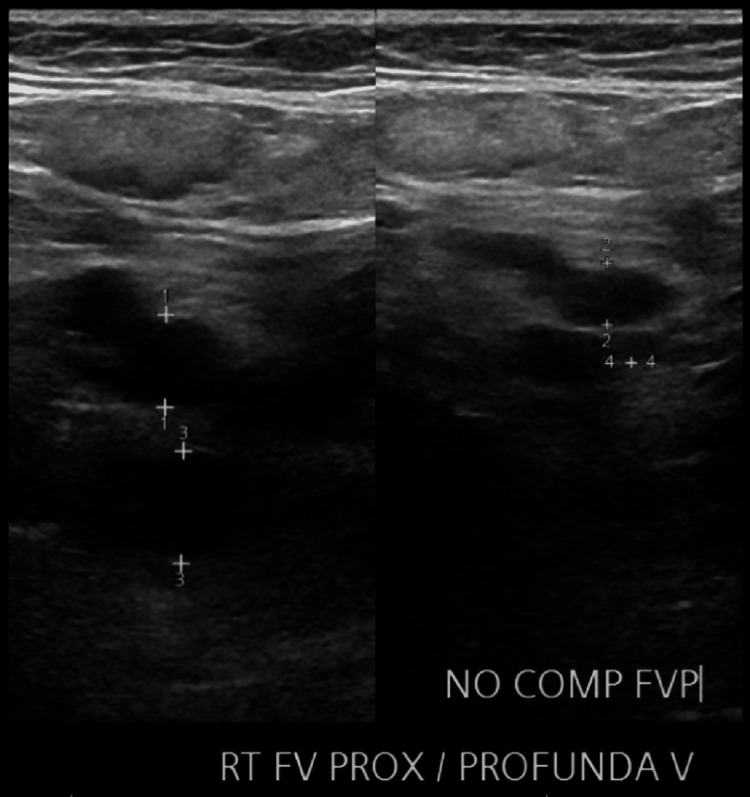
Right femoral vein showing no compressibility. RT FV PROX/PROFUNDA V: right femoral vein proximal/profunda vein; NO COMP: no compressibility; FVP: femoral vein proximal

**Figure 3:**
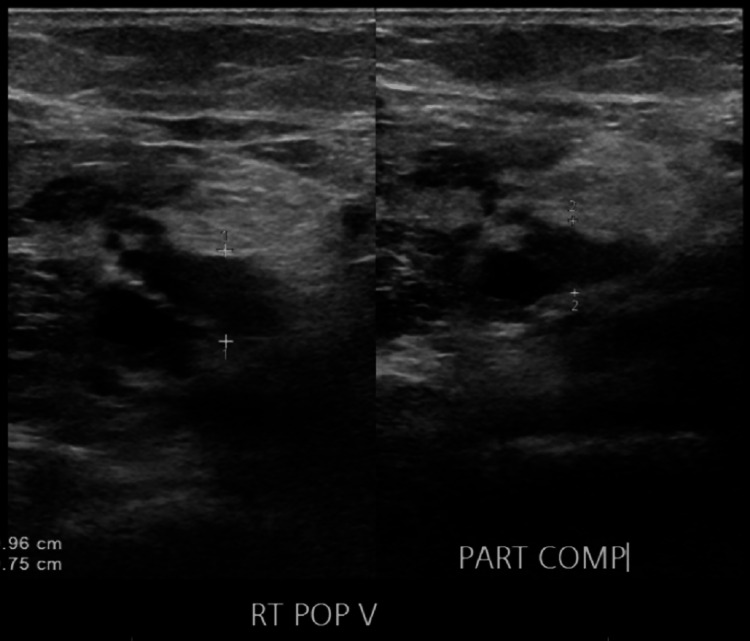
Right popliteal vein with partial compressibility. RT POP V: right popliteal vein; PART COMP: partial compressibility

Given the high clinical suspicion for acute PE, computed tomography angiography (CTA) of the chest was performed. This revealed an SPE with a clot burden extending into both the right and left main pulmonary arteries (Figure 1). Venous duplex ultrasound of the lower extremities also confirmed acute deep vein thrombosis (DVT) involving the right femoral and popliteal veins (Figure 2, Figure 3). 

Transthoracic echocardiography demonstrated preserved biventricular function with no evidence of right ventricular strain. Serum troponin and proBNP levels were within normal limits. These tests were obtained to evaluate for evidence of right heart strain given the diagnosis of pulmonary embolism.

The patient was admitted to the intensive care unit (ICU) and initiated on intravenous unfractionated heparin infusion, which was preferred over low-molecular-weight or direct oral anticoagulants due to its rapid titrability and ease of reversal in the event of clinical deterioration or need for invasive procedures. He required two days of close monitoring in the ICU, after which he was transferred to a general medical ward in stable condition. His anticoagulation regimen was transitioned from intravenous heparin to oral apixaban for long-term therapy.

Further laboratory work-up, including hypercoagulability testing, revealed normal protein C and protein S activity and no evidence of factor V Leiden mutation. This evaluation was performed after the patient had been discontinued from heparin therapy and downgraded from the ICU to avoid confounding effects on assay results. However, serum testing showed a markedly elevated homocysteine level of 134 µmol/L (reference range: 5-15 µmol/L) and a low vitamin B12 level of 142 pg/mL (reference range: 200-900 pg/mL).

The patient remained hemodynamically stable throughout hospitalization, required no advanced interventions, and had an otherwise uneventful clinical course. He was discharged home in stable condition on apixaban and vitamin B12 supplementation with outpatient hematology and primary care follow-up. The patient also received counseling on N₂O cessation and education regarding its potential complications.

## Discussion

This case highlights the association among N₂O abuse, hyperhomocysteinemia, functional vitamin B12 deficiency, and the development of severe venous thromboembolism (VTE), culminating in an SPE in a young adult without conventional risk factors. Reports of PE associated with N₂O remain rare but are increasing. Table 2 summarizes published cases, which consistently describe young adults presenting with severe hyperhomocysteinemia, thrombotic complications, and variable vitamin B12 levels associated with the recreational use of N₂O. 

**Table 2:** Summary of reported cases of pulmonary embolism in patients with recreational nitrous oxide abuse. LMWH: low-molecular-weight heparin; VA-ECMO: veno-arterial extracorporeal membrane oxygenation; F: female; M: male Reference value: homocysteine <15 μmol/L; vitamin B12 150-800 pmol/L, 160-950 ng/ml

Author, year	Age/sex	Clinical presentation	Lab findings	Site of thrombosis	Treatment	Outcome
Nguyen et al., 2024 [9]	42F	Dyspnea, chest/back pain	↑Homocysteine: 18.6 µM/L ↑B12: 1158 ng/ml	Bilateral PE	LMWH, Warfarin	Improved; no recurrence after N₂O cessation
Parein & Bollens, 2023 [10]	22F	Polyneuropathy, pancytopenia, dyspnea	↑Homocysteine: 207 µM/L ↓Vit B12: <148 ng/ml	Bilateral PE	Anticoagulation + B12 supplementation	Hematologic recovery, residual mild neuropathy
McMahon et al., 2024 [11]	21F	SOB, paresthesia, neuro deficits	↑Homocysteine: 78 µM/L, Vit B12: normal	SPE, cerebral infarcts, basilar artery thrombosis, limb thrombosis, splenic/renal infarcts	Anticoagulation, thrombectomy, B12 supplementation	Survived; residual neurological deficits
Vollenbrock et al., 2021 [12]	27M	Hypotension, hypoxemia	↑Homocysteine: 88 µM/L ↓Vit B12: 108 pmol/L	SPE, lung infarction	VA-ECMO, thrombolysis, embolectomy	Recovered
Pedersen et al., 2021 [13]	19 M	Peripheral neuropathy, muscle weakness, sensorial deficit, thoracic pain	↑Homocysteine: 92 µmol/L Vit B12: Normal	Bilateral central PE, pulmonary infarction	B12, B9, B1 supplementation, physiotherapy, rivaroxaban	Recovered, mild sensory-motor residual
Bizouard et al., 2024 [14]	23 M	Vomiting, diarrhea, febrile headache, and abdominal pain	↑Homocysteine: 51.9 μmol/L Vit B12 not reported	Right iliac venous thrombosis, bilateral PE	Enoxaparine, rivaroxaban	Recovered
Sun et al., 2019 [15]	29 M	Chest pain	↑Homocysteine: 24.12 µmol/L ↑ Vit B12: 734 pmol/L	Calf muscular venous thrombosis in the right extremity, Bilateral central PE	Alteplase, anticoagulant therapy, B9	Recovered
Caris et al., 2023 [16]	20 F	Syncopal event after 3 weeks of progressive dyspnea.Tachypnea, fever, tachycardia, oxygen saturation level below 90%, tight right calf	↑Homocysteine: 253 μmol/L ↓ Vit B12 142 pmol/L	DVT, bilateral segmental and subsegmental PE, pulmonary infarction.	LMWH, rivaroxaban, B12 supplementation	Recovered

Collectively, these reports emphasize several important patterns. First, most patients are otherwise healthy young adults, frequently without conventional thromboembolic risk factors. In some cases, minor additional risks such as smoking or oral contraceptive use were present, but they alone could not account for the severity of events observed [11,12]. 

Second, hyperhomocysteinemia was a nearly universal finding, even in patients with normal or elevated serum vitamin B12 levels, underscoring that functional inactivation of methylcobalamin is not reliably detected by standard assays [9,11,12]. Third, the vascular manifestations are strikingly heterogeneous, ranging from PE and cerebral venous thrombosis to severe multivessel arterial occlusion.

Pathophysiology

The pathophysiological link between N₂O use and thrombosis is mediated through the disruption of vitamin B12-dependent pathways. 

Vitamin B12 normally functions in two active forms: methylcobalamin, a cofactor for methionine synthase, and adenosylcobalamin, a cofactor for methylmalonyl-CoA mutase [17,18]. N₂O irreversibly oxidizes the cobalt atom in methylcobalamin, rendering it inactive and producing a functional B12 deficiency despite normal or elevated serum levels [19,20].

This inactivation blocks methionine synthase, preventing the conversion of homocysteine to methionine and impairing tetrahydrofolate recycling, leading to profound hyperhomocysteinemia. Elevated homocysteine promotes endothelial dysfunction, platelet activation, and resistance to fibrinolysis, creating a potent prothrombotic environment [17]. In parallel, reduced methionine and S-adenosylmethionine impair DNA synthesis and methylation, resulting in hematologic abnormalities such as megaloblastic anemia and in defective myelin formation underlying neurological complications [19,21]. 

**Figure 4:**
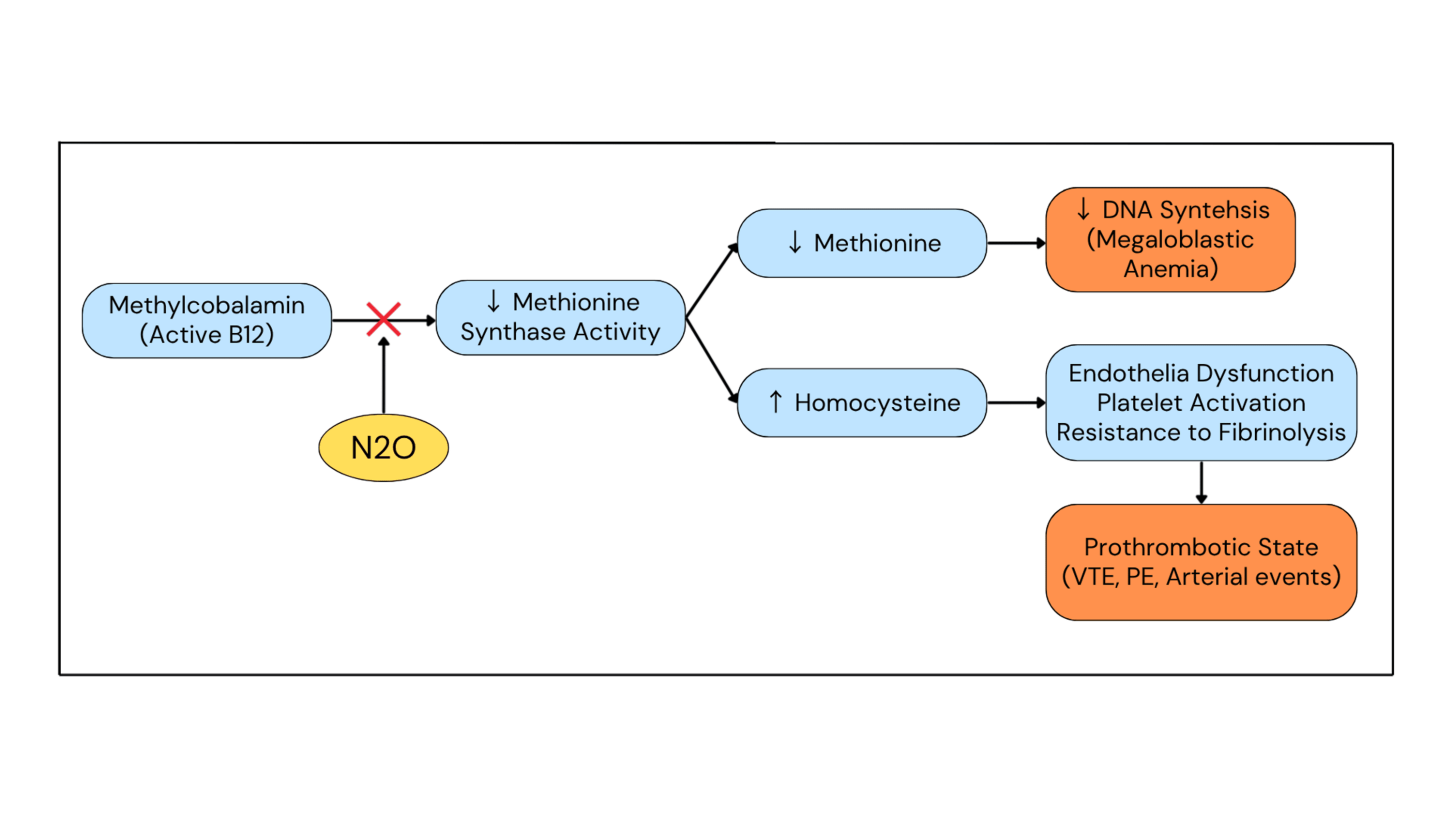
Mechanism of nitrous oxide-induced thrombosis via vitamin B12 inactivation. N_2_O irreversibly oxidizes the cobalt atom of methylcobalamin, the active form of vitamin B12, leading to functional B12 deficiency. This inhibits methionine synthase, blocking the remethylation of homocysteine to methionine. As a result, homocysteine accumulates, producing endothelial dysfunction, platelet activation, and resistance to fibrinolysis, all of which contribute to a prothrombotic state. Reduced methionine impairs DNA synthesis, leading to megaloblastic anemia. Original schematic created by the authors

Prolonged N₂O use also causes secondary depletion of adenosylcobalamin, with accumulation of methylmalonic acid that further disrupts fatty acid metabolism and myelin stability. Collectively, these derangements explain how N₂O induces a prothrombotic and neurotoxic state, even in patients whose total serum B12 concentrations appear normal. Figure 4 better represents the mechanism by which N₂O induces thrombosis via vitamin B12 inactivation.

Management strategies

Reported cases have shown that treatment generally involves anticoagulation, vitamin B12 supplementation, and discontinuation of N₂O use [9-12,16]. Anticoagulation with heparin, vitamin K antagonists, or direct oral anticoagulants is essential for controlling the acute thrombotic event, while high-dose parenteral vitamin B12 therapy restores methylcobalamin activity and helps normalize homocysteine levels [9-12]. Complete cessation of N₂O use is critical, as supplementation alone is ineffective if exposure persists [9, 10, 16]. Most patients have favorable outcomes with anticoagulation and vitamin B12 therapy, emphasizing the importance of addressing both the acute thrombotic process and the underlying metabolic disturbance.

Outcomes

Our review also highlights variability in outcomes. Patients who presented with isolated PE generally recovered with anticoagulation and cessation of N₂O use. Conversely, cases involving extensive arterial thrombosis often required invasive interventions such as thrombectomy or extracorporeal membrane oxygenation, with residual neurological or functional deficits in survivors. This spectrum of severity emphasizes the importance of early recognition and prompt management.

Clinical implications

Early recognition of N₂O abuse as an emerging cause of VTE in young adults without traditional risk factors has direct implications for clinical practice. Since it is not commonly considered in the differential diagnosis, its recognition may be delayed, thereby complicating the clinical approach. Nevertheless, N₂O exposure should be considered during the initial evaluation of unexplained thrombotic events in young patients, enabling timely diagnosis and comprehensive management. A targeted history is crucial, and clinicians should maintain a high index of suspicion in patients with unusual thrombotic presentations, especially when traditional risk factors are absent and laboratory testing reveals hyperhomocysteinemia.

## Conclusions

N₂O can induce a functional vitamin B12 deficiency, leading to elevated homocysteine levels and increased thrombotic risk. This case underscores the importance of evaluating vitamin B12 and homocysteine levels in patients with unexplained thromboembolic events and considering N₂O exposure as a reversible cause of hyperhomocysteinemia. Early initiation of anticoagulation and counseling on cessation of N₂O use are essential for effective management and prevention of recurrence. Although this report describes a single case and has limited generalizability, understanding this mechanism may help guide targeted therapies and prevent future thromboembolic episodes. Further studies are needed to establish appropriate screening protocols, develop risk stratification tools, and determine optimal long-term management strategies for young patients presenting with thromboembolism and vitamin B12 deficiency.
